# COVID-19 Infection May Not Produce Lasting Immunity in Non-vaccinated Young Adults: A Retrospective Review of COVID-19 Antibody Data

**DOI:** 10.7759/cureus.23698

**Published:** 2022-03-31

**Authors:** Marisa B DiGiulio, Rajia Arbab, Kevin P Driscoll, John Balmer DO

**Affiliations:** 1 Family and Community Medicine, Lake Erie College of Osteopathic Medicine, Erie, USA; 2 Family Medicine, Spartansburg Regional Health Center & Canadohta Lake Health Center, Spartansburg, USA

**Keywords:** covid severity, sars-cov-2-igg antibodies, covid, covid-19, covid-19 prevention, covid-19 spread, recurrence covid 19, immunology covid 19, covid-19 pandemic, covid-19 vaccine

## Abstract

Background

Despite progress in achieving herd immunity through recovery from previous infection and vaccination efforts, the COVID-19 pandemic continues to be an imminent health concern. Exposure to the severe acute respiratory syndrome coronavirus 2 (SARS-CoV-2) viral antigen through infection or vaccination facilitates immune system efficacy against future infection, but it is currently unclear how long this immunity lasts. Therefore, understanding the necessary exposures to produce adequate antibody levels and the duration of this humoral response to prevent infection is imperative in updating guidelines for vaccination and ultimately ending this public health crisis.

Aims

This study aimed to compare the presence of serum antibodies in younger and older age groups to determine how vaccination and previous infection compare as indicators of immunity against COVID-19. We also evaluated age to determine its role in antibody presence. We hope that this information will be helpful to the public to develop the best recommendations for vaccination guidelines concerning distinct demographics. ​

Materials and methods

In this retrospective data analysis, we evaluated saliva SARS-CoV-2 test results taken from 309 subjects (192F/117M; median age=53.4) during a community fair in Crawford County, PA. We sorted the subjects into groups based on age, reported infection with the COVID-19 virus, and vaccination status. We then performed a Chi-square analysis to compare the frequency of positive SARS-CoV-2 antibody tests within these groups.

Results

The vaccinated but not previously-infected cohort (n=146, 81.5%) was significantly more likely to have antibodies than the unvaccinated infected cohort (n=55, 65.5%; p<0.0001). In the previously-infected, unvaccinated cohort, individuals who were 55 and older were more likely to have antibodies than younger individuals (p<0.0157), but no age-dependent difference was observed among vaccinated individuals.

Conclusions

The results suggest that vaccination provides a more durable immune response than recovery from infection, and there is an age-dependent humoral response following previous infection but not vaccination. Practically speaking, this information implies that despite popular misconception, individuals under the age of 55 must receive a COVID-19 vaccine despite the previous infection as they are significantly less likely to have antibodies following infection than their counterparts who are over the age of 55.

## Introduction

With more than 413 million reported cases, the COVID-19 pandemic has resulted in more than five million deaths worldwide [1]. Given the devastating nature of the pandemic, determining immune responsiveness is integral to decreasing virus-associated mortality and hospitalization. One measure of immune system recognition and response to the virus is circulating antibodies specific for SARS-CoV-2. In this study, antibodies to the spike protein were assessed as they are a measure of humoral immunity following both recovery from COVID-19 and vaccination for SARS-CoV-2.   

It is widely accepted that COVID-19 recovery and vaccination result in SARS-CoV-2 neutralizing antibodies, which prevent reinfection, but these antibodies decrease over time [2]. Given the ephemeral nature of SARS-CoV-2 antibodies in circulation [3], the misconception that previous illness allows for indefinite immunity is harmful as it misleads many to neglect vaccination and social distancing practices. 

As suggested by previous studies, there is a great deal of heterogeneity in the durability and duration of humoral immunity to the virus, and this largely depends on the subject population and severity of symptoms.  A study examining 111 patients with confirmed cases of COVID-19 suggests that hospitalized patients with longer symptom duration had higher levels of antibody neutralizing activity than the antibodies of non-hospitalized individuals [4]. Furthermore, younger individuals with asymptomatic or milder infections by SARS-CoV-2 induce a weaker immune response than those with more severe infections [5,6].  In these less severe cases, this results in lower production of antibodies and a consequently shorter-term resistance against reinfection. This suggests that younger, asymptomatic populations may have lower quantities and efficacies of antibodies as compared to their older counterparts.  

Pertinent to this present study, it is well-documented that younger populations tend to be less vaccinated in the United States (Table 1) [1].  This immunization hesitancy among younger individuals has prevailed since the beginning of SARS-CoV-2 vaccine availability, even in areas of high vaccination acceptance, such as Connecticut, which had 66.71% of its population fully vaccinated as of September 11, 2021 [7,8]. This raises concern as vaccine hesitancy in combination with shorter-term immunity due to less severe symptoms in younger populations may make them more vulnerable to infection, serving as a nidus to greater rates of viral transmission.  

The aim of this study was to examine younger (<55 years, n=150) as well as older age groups (≥ 55 years, n=158) to determine the efficacy of vaccination and previous infection in producing antibodies and thereby preventing infection. This understanding will help healthcare providers choose the best recommendations for individual patients and government officials to develop the best vaccination guidelines for the public. 

## Materials and methods

Review protocol

A retrospective analysis of medical records from 309 attendees who presented for testing for SARS-CoV-2 antibodies at the Spartansburg Community Fair in Crawford County, Pennsylvania, from September 6-11, 2021, was conducted. Information required by the Pennsylvania Department of Health was collected. This information included name, address, date of birth, age, gender, race, ethnicity, previous COVID-19 infection, and vaccine status. The required information and the test results were reported to the Department of Health as required by law. Each participant was a current patient or was accepted as a patient of Dr. Balmer. Paper medical records were created for each patient at the event and were later scanned in the patient’s electronic medical record system. 

Identifying patient information was permanently removed from the paper record by the physician’s staff. The primary author of this paper extracted and analyzed the remaining data. Patient protection utilized during the retrospective chart review was approved for IRB exemption by the Lake Erie College of Osteopathic Medicine Institutional Review Board and by the HIPPA compliance officer of John E. Balmer, D.O. 

Testing process

The SARS-CoV-2 antibody testing was ordered in appropriate patients and performed by office staff using the CovAb test (Diabetomics, Inc., Hillsboro, OR, USA). The FDA made this test available for Emergency Use Authorization (EUA) on June 4, 2021. Gingival crevicular fluid (GCF) samples were taken from the gum line of each subject and tested for the presence of SARS-CoV-2 antibodies with test cartridges manufactured by Diabetomics.  This saliva-based sampling method detected Ig antibodies to the spike protein, which is a component of the surface of the SARS-CoV-2 virus envelope. It detects all three classes of antibodies (IgA, IgG, IgM) and has been tested for cross-reactivity by the manufacturer against many other major viruses, including but not limited to influenza virus, HIV, and enterovirus. The CovAb test has been reported to yield a sensitivity of 97.6% and a specificity of 98.8% when used on individuals at least 14 days following the onset of symptoms.  

Analysis

Subjects were sorted into groups based on reported infection with the COVID-19 virus and vaccination status. To distinguish the efficacy of humoral immunity due to previous infection and vaccination, Chi-square tests were performed to compare the frequency of positive CoVAb antibody tests among vaccinated and previously infected groups. Additionally, Chi-square tests were conducted to assess the frequency of positive test results among different age groups and vaccine manufacturers. P-values less than 0.05 were considered significant.

## Results

On September 11, 2021, the population group in this study had a vaccination rate of 56.0% which mimics the rates of vaccination in Pennsylvania (56.2%) and the United States (54.0%) [1]. Similar to vaccination rates in the United States as a whole, this study's population has greater vaccination rates with increasing age (Table 1). 

**Table 1:** Percentage of fully-vaccinated citizens in the entire US and this study's population separated by age groups. This snapshot of vaccination rates was taken on September 11, 2021, the final day of data collection.

Age Range (years old)	US Percent (%) Fully Vaccinated on 9/11/2021	Study Population Percent (%) Fully Vaccinated
16-24	55.5	21.4 (n=14)
25-39	60.1	30.8 (n=52)
40-40	68.4	47.1 (n=51)
50-64	75.8	51.4 (n=105)
65-74	88.0	86.4 (n=66)
75+	82.8	90.4 (n=21)

In this study's population of 309 subjects, 51.1% (158) were 55 or older, and 49.9% (151) were younger than 55. In terms of gender, 37.9% (117) were male and 62.1% (192) were female. There was no significant difference in frequency of positive antibody results (p=0.192) or rate of vaccination (p=0.554) based on gender. Due to 100% of the sample identifying as white, we were unable to evaluate race as a variable (Table 2).

**Table 2:** Demographic characteristics of 309 attendees of the Spartansburg Fair who volunteered to participate and undergo SARS-CoV-2 antibody testing The total number of individuals endorsing/denying previous COVID-19 infection is only 303 due to 6 respondents declaring their status “Unknown”. J&J: Johnson & Johnson, +: Positive, - : Negative

Characteristic	Value
Mean age of subjects (n=309)	53.41 years old
Percentage of subjects ≥55	51.1% (158)
Percentage of subjects <55	49.9% (151)
Percentage of Males	37.9% (117)
Percentage of Females	62.1% (192)
Percentage with previous COVID-19 infection	26.4% (80)
Percentage who denied the previous infection	73.6% (223)
Percentage Vaccinated	56.0% (173)
Percentage Unvaccinated	44.0% (136)
# Vaccinated with Pfizer	78
# Vaccinated with Moderna	85
# Vaccinated with J&J	10
Percentage of (+) Antibody results	61.0% (189)
Percentage of (-) Antibody results	39.0% (120)

Of the 309 subjects, 76 reported neither being infected with the virus nor receiving a vaccine. Of these 76 individuals, only 14.5% (11) tested positive for antibodies. We used this group to represent the frequency of positive tests in those without known exposure to the viral antigen in order to control for false positives. We then compared this group’s rate to the frequency of positive antibody tests of those infected with the virus, but were vaccinated and those who were vaccinated but were not infected.  

**Figure 1:**
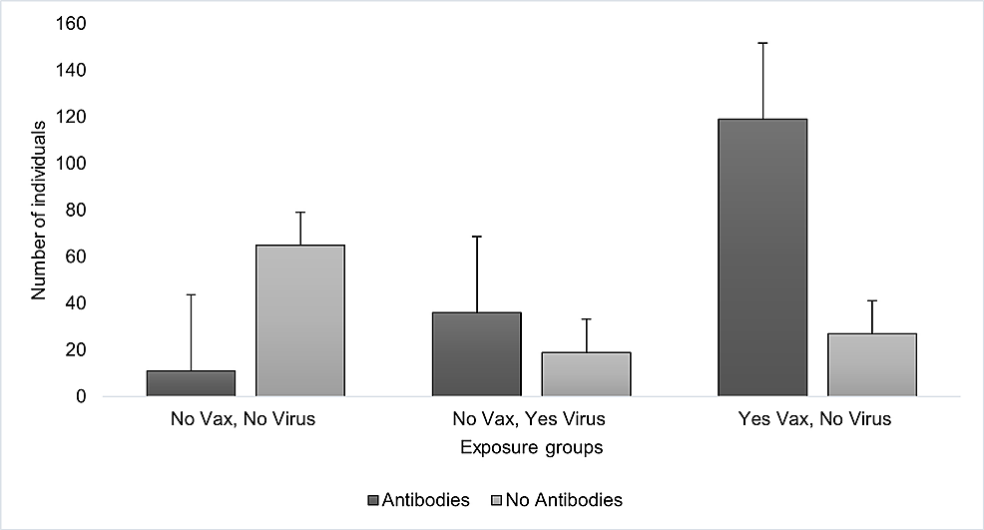
Frequency of positive (+) and negative (-) antibody tests based on exposure This graph displays the frequency of positive COVID-19 antibody test results in subjects based on vaccination status and previous infection with COVID-19. The presence of antibodies was determined by the administration of the Diabetomics CovAb SARS-CoV-2 Ab Test. Vaccination status and prior infection with COVID-19 were based on a survey given to subjects prior to antibody test administration. Comparing the frequency of positive and negative tests between groups was done by conducting a Chi-square analysis. (Vaccinated group to infection group p<0.0001, vaccinated group to neither p<0.0001, infection group to neither p<0.0001)

In the previously infected but not vaccinated cohort (n=55), 65.5% tested positive for antibodies which are significantly greater than the control (p<0.0001). In the vaccinated but not previously infected cohort (n=146), 81.5% tested positive for antibodies, significantly greater than the control (p<0.0001) and the previously infected but not vaccinated cohort (p=0.0157) (Figure 1).  

**Figure 2:**
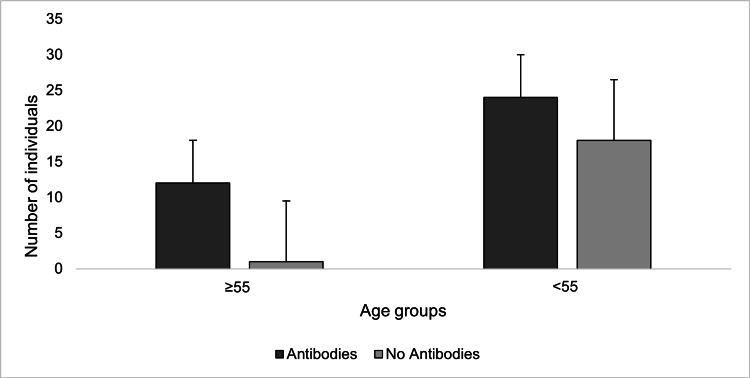
Frequency of positive (+) and negative (-) antibody tests in non-vaccinated, infected individuals vs. age This graph displays the frequency of positive COVID-19 antibody test results in subjects of two age groups with prior COVID-19 infection who did not receive a vaccine. The presence of antibodies was determined by the administration of the Diabetomics CovAb SARS-CoV-2 Ab Test. Vaccination status and prior infection were self-reported and based on a survey given to patients prior to antibody test administration. Comparing the frequency of positive and negative tests between groups was done by conducting a Chi-square analysis. (p-value= 0.0157)

It was found that individuals 55 and older were more likely to have antibodies than those younger than 55 (p=0.00278), regardless of vaccination status or previous infection. Additionally, this older cohort (≥55) has a higher rate of vaccination (74.7%) than the younger cohort (<55, 37.3%; p<0.0001). In the vaccinated but not previously infected cohort, age did not play a significant role in detected antibodies (p=0.191) (Table 3). However, in the previously infected but unvaccinated cohort, the older subjects (≥55) were significantly more likely to have detected antibodies than the younger subjects (<55, p=0.0157) (Figure 2). A comparison of differences in percentage of positive tests based on age within different exposure groups is displayed in Table 3.

**Table 3:** Percentage of subjects who tested positive for antibodies based on age, vaccination status, and previous infection with the virus

Groups	% positive for antibodies	
Not Vaccinated + No Infection	All: 14.5%	≥55: 14.8%
		<55: 14.2%
Not Vaccinated + Infected	All: 65.5%	≥55: 92.3%
		<55: 57.1%
Vaccinated + No Infection	All: 81.0%	≥55: 77.6%
		<55: 89.6%

## Discussion

Given the desire to establish herd immunity in the continuing pandemic, younger populations play a pivotal role in decreasing transmission of COVID-19 and thereby decreasing virus-related mortality and hospitalization rates. Our study is particularly relevant as it corroborates with earlier studies suggesting that younger individuals may have lower rates of neutralizing antibodies against COVID-19, and thus there is a greater public health incentive to vaccinate younger populations.  

Individuals younger than 55 have lower rates of vaccination, making them more susceptible to infection (as seen above in Table 2). Younger people often attribute this hesitancy to requiring more information about vaccine efficacy and safety [9]. One study of university students found that those who reported insufficient knowledge of vaccine safety were 6.061 times (p<0.001) more likely to be vaccine-hesitant. Students who had the misconception that immunity post-infection was superior to immunity achieved through vaccination were 3.347 times (p<0.001) more likely to be vaccine-hesitant [10]. Alternatively, the results of this study support the superior efficacy of vaccination compared to the previous infection. 

A contribution to this hesitancy may be misinformation regarding vaccination may be a reliance on social media for information regarding SARS-CoV-2. A study found that a sample of university students who depend on social media for information were 3.086 times (p<0.001) more likely to be vaccine-hesitant [10]. Because strengthening populations’ immune systems to SARS-CoV-2 is integral to the management of the COVID-19 pandemic, determining a way to relay accurate information to younger populations is necessary. Specifically, as demonstrated by the results of this study, the superior efficacy of vaccination compared to the previous infection must be emphasized.  

Our study has several limitations as there are several indicators of immunity other than antibody levels. These include other factors such as immune status, infection severity, cross-immunity, age, and other immunological factors such as T-cell and B-cell memory or lack of antibody neutralizing capacity [11-13]. Still, like humoral immunity, cell-mediated immunity also declines as SARS-CoV-2-specific CD4+ T cells and CD8+ T cells decline with a half-life of three to five months. While antibody detection should not be interpreted as absolute proof of resistance to SARS-CoV-2, it is a practical measure of humoral immune response to COVID-19 infection. Due to the nature of data collection, self-reporting bias in regards to the reporting of prior vaccination and infection is another possible limitation of the study.

## Conclusions

The study suggests that there is a great deal of heterogeneity regarding the strength of humoral response to SARS-CoV-2, particularly among younger populations. As antibodies decline following the infection, the long-term immune response may not be sufficient, and younger people with decreased antibodies following infection are particularly susceptible to reinfection. Additionally, because of the shorter duration and potency of this immune response among younger individuals, younger populations may even need to be vaccinated sooner after infection than their older counterparts. To properly assess the efficacy of the humoral immune response to SARS-CoV-2, there is a need for longitudinal studies to detect the levels of antibodies in vaccinated and previously-infected patients among different patient demographics at this point in the pandemic. This would account for complicating factors such as novel virus strains and age in order to establish updated policies regarding vaccines and vaccine booster administration. In conclusion, the younger population should be provided with accurate information about immunity and vaccination to encourage vaccine acceptance.
